# Early Biological Response to Poly(ε-Caprolactone)/Alumina-Toughened Zirconia Composites Obtained by 3D Printing for Peri-Implant Application

**DOI:** 10.3390/polym16172521

**Published:** 2024-09-05

**Authors:** Riccardo Pedraza, Alessandro Mosca Balma, Ilaria Roato, Clarissa Orrico, Tullio Genova, Giacomo Baima, Giovanni Nicolao Berta, Andrea Giura, Luigi Ribotta, Donatella Duraccio, Maria Giulia Faga, Federico Mussano

**Affiliations:** 1 Bone and Dental Bioengineering Laboratory, CIR Dental School, Department of Surgical Sciences, University of Turin, 10126 Turin, Italy; riccardo.pedraza@polito.it (R.P.); alessandro.moscabalma@unito.it (A.M.B.); ilaria.roato@unito.it (I.R.); clarissa.orrico@polito.it (C.O.); giacomo.baima@unito.it (G.B.); 2 Institute of Sciences and Technologies for Sustainable Energy and Mobility, National Council of Research, Strada delle Cacce 73, 10135 Turin, Italy; donatella.duraccio@stems.cnr.it (D.D.); mariagiulia.faga@stems.cnr.it (M.G.F.); 3 Department of Mechanical and Aerospace Engineering, Politecnico di Torino, Corso Duca degli Abruzzi 24, 10129 Turin, Italy; 4 Department of Life Sciences and Systems Biology, University of Turin, Via Accademia Albertina 13, 10123 Turin, Italy; tullio.genova@unito.it; 5 Department of Clinical and Biological Sciences, University of Turin, Regione Gonzole 10, 10043 Orbassano, Italy; giovanni.berta@unito.it; 6 Applied Metrology and Engineering Division, Istituto Nazionale di Ricerca Metrologica (INRiM), Strada delle Cacce 91, 10135 Turin, Italy; a.giura@inrim.it (A.G.); l.ribotta@inrim.it (L.R.)

**Keywords:** poly(ε-caprolactone), alumina-toughened zirconia, solvent casting, 3D printing, cell viability, early cell response, oral keratinocytes, oral fibroblasts

## Abstract

The improvement of the mucosal sealing around the implant represents a challenge, one that prompted research into novel materials. To this purpose, a printable poly(ε-caprolactone) (PCL)-based composite loaded with alumina-toughened zirconia (ATZ) at increasing rates of 10, 20, and 40 wt.% was prepared, using a solvent casting method with chloroform. Disks were produced by 3D printing; surface roughness, free energy and optical contact angle were measured. Oral fibroblasts (PF) and epithelial cell (SG) tests were utilized to determine the biocompatibility of the materials through cell viability assay and adhesion and spreading evaluations. The highest level of ATZ resulted in an increase in the average roughness (S_a_), while the maximum height (S_z_) was higher for all composites than that of the unmixed PCL, regardless of their ATZ content. Surface free energy was significantly lower on PCL/ATZ 80/20 and PCL/ATZ 60/40, compared to PCL and PCL/ATZ 90/10. The contact angle was inversely related to the quantity of ATZ in the material. PF grew without variations among the different specimens at 1 and 3 days. After 7 days, PF grew significantly less on PCL/ATZ 60/40 and PCL/ATZ 80/20 compared to unmixed PCL and PCL 90/10. Conversely, ATZ affected and improved the growth of SG. By increasing the filler amount, PF cell adhesion and spreading augmented, while PCL/ATZ 80/20 was the best for SG adhesion. Overall, PCL/ATZ 80/20 emerged as the best composite for both cell types; hence, it is a promising candidate for the manufacture of custom made transmucosal dental implant components.

## 1. Introduction

Over the last few decades, dental implants have emerged as the preferred treatment option for rehabilitating edentulism, since they represent a reliable and enduring solution which can be used to reestablish the aesthetics and function of natural teeth, even with immediate loading protocols [1]. Also, the neoformation of bone tissue in continuity with implant fixtures—known as osseointegration—prevents post-extractive jaw bone resorption [2]. Most dental implant systems on the market are characterized by roughened titanium surfaces, which have been shown in the literature to promote osseointegration [3]. These surfaces have been pointed out, albeit not unanimously [4], as favorable environments for biofilm accumulation, and hence the onset of peri-implantitis [5], i.e., the progressive bone loss around the fixture associated with the microbial colonization.

Owing to the high prevalence of peri-implantitis [6], researchers oriented their studies to the prevention of this disease, using a two-fold approach by introducing antibacterial surfaces on the intraosseous fixtures [7] and enhancing the adhesion of the peri-implant tissues [8]. Attaining a strong mucosal seal of the abutments, i.e., the transmucosal components of the implants, has become, therefore, paramount in order to prevent implant failure [9]. Promising results were achieved, for instance, with a Poly(dopamine)-modified alkali-heat-titanium surface enriched with hydroxyapatite and carboxymethyl chitosan, as it could enhance human gingival fibroblast adhesion, spread and proliferation, also ensuring antibacterial activity [10]. Even anodization of titanium has seemed beneficial for reducing the colonization of representative bacterial strains [11], while having remarkable effects on fibroblast proliferation only at the nanoscale [12].

These approaches tackled only the connective component, neglecting the epithelium, which is the outermost barrier between the organism and the environment. Yet the peri-implant epithelium, usually named the “long junctional epithelium” [13], results from the downward migration of the oral epithelium parallel to the abutment, which is only stopped by the connective tissue with its circumferential collagen fibers surrounding the abutment [14]. Lacking perpendicular fibers, the peri-implant connective tissue is not as tightly adherent as the periodontal connective tissue, and thus it may allow the junctional epithelium to penetrate too deeply, resulting ultimately in bone loss [15]. A key achievement of the research would be to develop a bio-interface capable of promoting an improved biomimicry of the normal periodontium.

Such an ambitious goal would entail the re-formation of an internal basal lamina between the junctional epithelium and the abutment, capable of limiting the former’s downward migration. To this purpose, among dental implant biomaterials [14], alumina-toughened zirconia (ATZ) appears as a promising option based on its peculiar capacity of orienting laminin 332, which is indispensable for guiding proper epithelial cell adhesion [16]. Besides this remarkable result achieved in vitro, ATZ could also outperform titanium when used as an implant material in a minipig model [17]. As a massive ceramic material, however, ATZ is hindered by its poor mechanical properties [18]. For this reason, combining the bioactivity of this material with polymers which are endowed with high tenacity although almost biologically inert, like UHMWPE, has recently become a topic of interest [19].

Among the numerous polymers available for regenerative medicine, the Poly(ε-caprolactone) (PCL) holds particular interest, being a biocompatible, synthetic, aliphatic polyester that, besides a widely diffused application for intraosseous scaffolds [20], has more recently revealed a remarkable suitability in periodontal ligament regeneration [21]. After proper manipulation [22] PCL could indeed guide fibroblast adhesion. This feature, together with its use as epidermal equivalent [23], and its versatility in the preparation of bioresorbable coatings [24], renders PCL the candidate of choice for preparing fibro-mucosal interfaces along the abutments.

Recently, our group obtained PCL-ATZ composites through solvent casting with better results than obtained with the dry mixing technique [25], paving the way to the easy tuning of the mechanical, and possibly biological, properties of these biomaterials. In the present study, the authors characterized in vitro the biological responses of three different ATZ/PCL compounds, with respect to oral fibroblasts and keratinocytes, in established cell models of gingival tissue. The purpose of this research was to assess whether the cell response could be modulated by simply varying the concentration of ATZ within the PCL matrix, with the aim of selecting a formulation possibly useful for the fabrication of functionalized implant abutments to ameliorate difficulties in the peri-implant mucosal seal.

## 2. Materials and Methods

### 2.1. Sample Preparation

An ester-terminated polycaprolactone (CAS-n 24980-41-4) matrix (CELLINK PCL TP-60505, Bico Group, Gothenburg, Sweden) was charged with alumina-toughened zirconia (ATZ, made of 20 wt.% Al_2_O_3_ and 80 wt.% 3Y-TZP composed of 3 mol% yttria-stabilized zirconia, Tosoh Bioscience, Tokyo, Japan) through solvent casting with chloroform (CHCl_3_, CARLO ERBA Reagents s.r.l., Cornaredo, Italy), as previously described [25]. Three PCL/ATZ composite materials were prepared: PCL/ATZ 90/10, PCL/ATZ 80/20, and PCL/ATZ 60/40. Unmixed PCL was considered as a control.

Three-dimensional planar samples were printed using a thermoplastic pneumatic printhead with a BIO X 3D bioprinter (CELLINK Bico Group, Gothenburg, Sweden). A standard square base geometry of 15 mm × 15 mm with a thickness of 0.65 mm and a 100% infill was printed directly on the glass surface of a Petri dish in order to obtain the smoothest interaction surface possible for each compound. These samples were used for surface roughness and contact-angle measurements. Cylindrical discs were obtained by cutting the previously described square 3D printed geometry with a 6mm biopsy punch, and were used for protein adsorption, cell adhesion, cell spreading, and cell viability tests, as well as SEM analysis. The print-bed temperature was set at 30 °C, with a clean chamber fan kept on. Printhead temperature was 115 °C for unmixed PCL, 125 °C for PCL/ATZ 90/10, 135 °C for PCL/ATZ 80/20, and 145 °C for PCL/ATZ 60/40. A nozzle 0.4 mm in diameter was employed; the imposed pressure was 190 kPa, and the printing speed was set at 2 mm/s.

### 2.2. Microscopy

Microstructure was studied by means of a Scanning Electron Microscope (Phenom XL G2 Desktop SEM, Thermo Fisher Scientific, Waltham, MA, USA). Before examination, the samples were: (a) washed in distilled water; (b) rinsed thoroughly in 70% ethanol–water solution; (c) cleaned ultrasonically in absolute ethanol for 20 min; (d) air dried under a chemical hood; and (e) coated with a thin conductive layer of gold. The instrument settings adopted for micrographs and analyses were 10 kV of voltage v in a MAP configuration with a Back Scatter Detector (BSD).

### 2.3. Roughness

Surface roughness was calculated according to ISO 21920 (for profile R parameters) and ISO 25178 (for areal S parameters) on unmixed PCL, PCL/ATZ 90/10, PCL/ATZ 80/20, and PCL/ATZ 60/40 samples; the analysis was carried out by both contact-based and optical profilometers. The contact-based test was made with a stylus profilometer (Form Talysurf PGI Novus S 10, Taylor Hobson Limited, Leicester, UK) equipped with a precision ceramic ball, which is calibrated by an interferometric setup to be traceable to the International Systems of Unit (SI). During the measurements, a cut-off of 0.08 mm was employed, and a total length of 8 mm was set. With the contact technique, roughness parameters Arithmetical Mean Height (R_a_), Root Mean Square Height (R_q_), Maximum Height (R_z_), Skewness (R_sk_) and Kurtosis (R_ku_) were calculated.

The second technique utilized the optical profilometer (Sensofar Plμ 2300, Barcelona, Spain) in confocal mode to perform the topography measurements. As before, the cut-off was set at 0.08 mm and the surface size was 254.64 μm × 190.90 μm, since a Nikon LU Plan Fluor 50×/0.80 objective was used. With this second technique, Arithmetical Mean Height (S_a_), Root Mean Square Height (S_q_), Maximum Height (S_z_), Skewness (S_sk_) and Kurtosis (S_ku_) surface parameters were measured; so not to ruin the specimen, optical measurements were performed before tactile measurements. For each sample, 5 optical measurements and 3 stylus measurements were carried out. The topographies and the profiles were processed by using MountainsMap Premium 10 by Digital Surf (Besançon, France).

### 2.4. Contact Angle and Surface Free Energy Evaluation

Surface wettability was assessed using a Biolin Scientific Theta Lite Optical Tensiometer (Stockolm, Sweden) using double-distilled water (dH_2_O) and diiodomethane (CH_2_I_2_). The contact angle was evaluated by the sessile drop method. For each liquid drop (1 μL in volume) dispensed, an image of the drop on the sample was acquired with the integrated high-resolution camera. The drop profiles were extracted and fitted with integrated software. At the liquid–solid interface, contact angles between fitted function and base line were calculated. For each sample and liquid probe, the contact-angle measurement was repeated five times on different areas.

The Owens–Wendt–Rabel–Kaelbele (OWRK) method was adopted to calculate a value of surface free energy, following the method proposed by Waldner, C. et al. [26]. Total (γ), polar (γ^P^), and dispersive (γ^D^) components were calculated by simple linear regression. Properties of dH_2_O and CH_2_I_2_ were taken as standard constants to perform the interpolation, as reported in Table 1 [27].

### 2.5. Protein Adsorption

To quantify the amount of protein adsorbed onto the samples, as described before [28], a 5% solution of Bovine Serum Albumin (BSA) in Phosphate Buffered Saline (PBS) was prepared and used to cover all the compounds. Specimens were incubated at 37 °C for 20 min, and then were washed twice with PBS. The total adsorbed protein amount was determined by means of elution with Tris Triton buffer (10 mM Tris (pH 7.4), 100 mM NaCl, 1 mM EDTA, 1mM EGTA, 1% Triton X-100, 10% Glycerol and 0.1% SDS) for 10 min, with the result subsequently quantified through Pierce™ BCA Protein Assay Kit (Life Technologies, Carlsbad, CA, USA) according to the manufacturer’s instructions.

### 2.6. Cell Experiments

#### 2.6.1. Cell Culture

Primary Palatal Fibroblasts (PF) and human gingival epithelioid cell line (SG) [29] were utilized to characterize the biological response in vitro, as induced by the different printed samples. PF were cultured in Alpha-MEM (Life Technologies, Milano, Italy), 10% fetal bovine serum (FBS, Life Technologies, Milan, Italy), and 5% penicillin (100 U/mL)-streptomycin (100 μg/mL) at 37 °C and with a 5% CO_2_ atmosphere. SG cells were maintained in an RPMI-1640 medium (Euroclone, Pero, Italy) with 10% FBS, 100 U/mL penicillin, and 100 μg/mL streptomycin.

#### 2.6.2. Cell Adhesion and Cell Spreading

Cells were seeded at 4000 cells/well on the samples with different percentages of filler and incubated for 40 min, then fixed by 4% paraformaldehyde solution, and washed with PBS; cell nuclei were stained by 1 μM DAPI (Merck, Darmstadt, Germany) for 15’ at 37 °C.

For the cell spreading evaluation, cells were maintained in culture for 24 h, then fixed and stained with Phalloidin (Cell Signaling technology, Danvers, MA, USA) for cytoskeleton and with DAPI for cell nuclei.

Images were acquired using a Nikon Eclipse Ti-E microscope with Nikon Plan 40×/0.75 and Nikon Plan 10×/0.10 objectives. Cell nuclei were counted using the ‘Analyze particles’ tool of ImageJ software (Version 2.14.0/1.54f, ImageJ, U. S. National Institutes of Health, Bethesda, MD, USA). For cell spreading analysis, 4 images were acquired at a “higher magnification” for each sample type in triplicate, and were firstly processed with the cellpose [30,31] cyto3 segmentation algorithm, and then pre-trained with other similar images in a controlled learning process, to obtain the contours of the single cell on all the acquired fields. Then, 12 different shape descriptors were measured for each detected cell with the Set Measurements function implemented in Fiji/ImageJ (i.e., area, perimeter, best fitting ellipse (BFE) major axis, BFE minor axis, BFE aspect ratio, BFE angle, circularity, roundness, solidity, Feret’s diameter, Feret’s angle, and minimum caliper diameter). From these 12 descriptors, 6 were selected as relevant parameters for cell morphology evaluation (area, perimeter, BFE aspect ratio, BFE angle, circularity, and roundness). Data were analyzed and plotted in MATLAB (MATLAB R2024a; The MathWorks, Inc., Natick, MA, USA).

#### 2.6.3. Cell Viability

PF and SG were plated on the different samples at a density of 10,000 cells/well in 96-well culture plates, in their cell culture media. Cell viability was assessed after 1, 3, and 7 days of in vitro culture through use of the Cell Titer GLO kit (Promega, Milan, Italy) according to the manufacturer’s protocol. Cell viability was expressed as relative light unit (RLU).

### 2.7. Statistical Analysis

Statistical analysis was performed through STATA software (version 18.0; StataCorp, College Station, TX, USA). One-way ANOVA was performed to evaluate differences among group variances at different time steps, and a Bonferroni post hoc corrective coefficient was adopted to find which groups differed in a statistically relevant way. Repeated *t*-tests were used to perform statistical analyses on shape descriptors for cell morphology evaluation. An α significance level of 0.05 was utilized [32,33].

## 3. Results

### 3.1. SEM Morphological Surface Analysis

**Figure 1:**
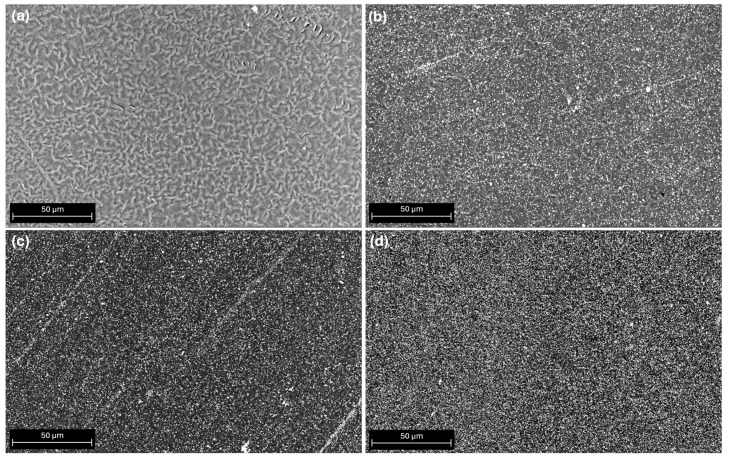
Scanning electron micrographs of materials: (**a**) unmixed PCL, (**b**) PCL/ATZ 90/10, (**c**) PCL/ATZ 80/20, and (**d**) PCL/ATZ 60/40.

Figure 1a shows the unmixed PCL surface, while PCL/ATZ 90/10, PCL/ATZ 80/20, and PCL/ATZ 60/40 surfaces are represented in Figure 1b, 1c, and 1d, respectively. In composites, the augmenting presence of ATZ filler is indicated by increasing numbers of white dots. Although all the samples were printed on the same glass support to obtain the smoothest and the most uniform possible surface, the ATZ distribution became more homogeneous proportionally to the increase of the amount of filler. Indeed, some residual stripes of ATZ aligned to the printing direction, likely due to the passage of the print head during the fused deposition process (Figure 1b,c), this disappeared when the highest ATZ percentage was used.

### 3.2. Roughness Analysis

PCL/ATZ 60/40 samples reached the highest roughness values, as reported from the averages Ra and S_a_ and the root-mean-square values R_q_ and S_q_ (Table 2 and Table 3, respectively). Skewness parameters (S_sk_) were approximately 1.5 for both unmixed PCL and PCL/ATZ 60/40, indicating an asymmetrical distribution of peaks and valleys in which more peaks are present than valleys. A large standard deviation was observed for these values due to the large exponent used for their calculation; indeed, parameters S_sk_ and S_ku_ are sensitive to the presence of spikes as well as narrow and deep valleys. However, the measurements were repeated to find whether there was a positive or negative tendency in the mean values. PCL/ATZ 80/20 showed values of Skewness that were near to 0, representing a homogeneous distribution of peaks and valleys which are very sharp in their profile, as highlighted by values of Kurtosis (S_ku_) greater than 3. All the samples were characterized by a leptokurtic distribution of valleys or peaks (S_ku_ > 3). All composites showed higher Maximum Height (S_z_) values than unmixed PCL, regardless of the ATZ amount.

### 3.3. Wettability and Surface Free Energy Evaluation

**Figure 2:**
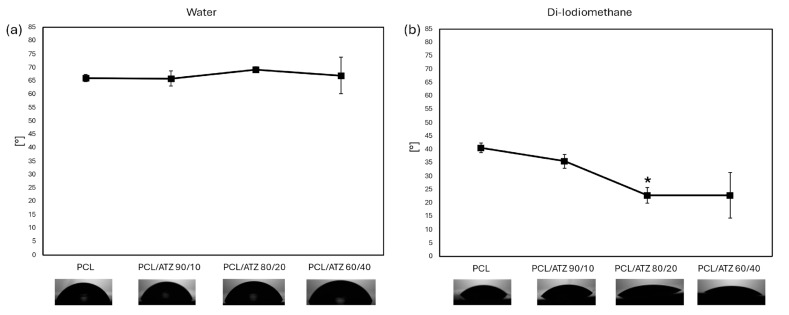
Graphs representing surface contact angle calculated in a hydrophilic environment (dH_2_O, (**a**)) and in a lipophilic environment (CH_2_I_2_, (**b**)) (* for *p* < 0.05).

The responses of the materials to both hydrophilic and lipophilic environments were assessed by returning to the standard optical contact angle (OCA) measurement with a polar solvent and an apolar solvent. The measured dH_2_O contact angles for all materials remained stable at approximately 67°, with no relevant differences found among the samples (Figure 2a). The only specimen that demonstrated a slight, but not significant, difference was PCL/ATZ 80/20 (Figure 2a). In a lipophilic environment with CH_2_I_2_, unmixed PCL showed the highest contact-angle value (41°), while all composite materials presented a decreased contact angle, which was 36° in PCL/ATZ 90/10 and 23° in both PCL/ATZ 80/20 and PCL/ATZ 60/40 (Figure 2b). The only statistically significant difference was detected between PCL and PCL/ATZ 80/20 (*p* < 0.05).

The SFE calculation showed higher values of total surface energy (γ) in the presence of the polymer alone (4.15) or with lesser ATZ filler (PCL/ATZ 90/10), and lower values of γ for PCL/ATZ 80/20 (4.02) and PCL/ATZ 60/40 (4.09). This behavior was also seen for the polar component of surface energy, although there were no significant variations in the values of dispersive surface energy, as shown in Table 4.

### 3.4. Protein Adsorption

**Figure 3:**
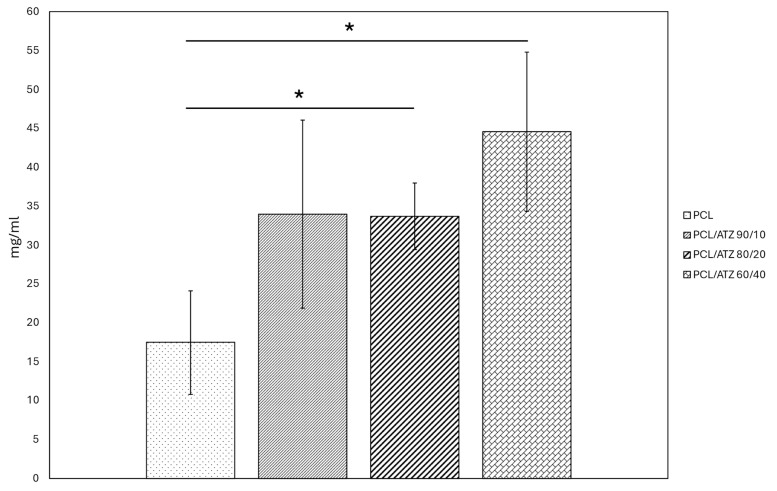
Graph representing the amount of BSA protein (mg/mL) adsorbed on specimen surfaces ((*) for *p* < 0.05).

The protein adsorption graph (Figure 3) shows that the amount of BSA protein adsorbed on the sample surfaces tended to increase with the ceramic filler percentage. Unmixed PCL displayed the lowest value (~15 mg/mL), compared to composites. In particular, PCL/ATZ 60/40 showed the highest value (~45 mg/mL) among all tested conditions with results significantly different from those of the pure polymer (*p* < 0.05). While PCL/ATZ 80/20 and PCL/ATZ 90/10 were similar in mean value, only the former differed from PCL in a statistically significant way (*p* < 0.05).

### 3.5. Cell Experiments

#### 3.5.1. Cell Adhesion and Cell Spreading

**Figure 4:**
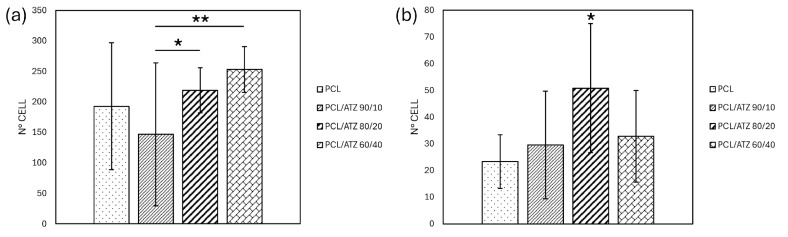
Graph representing PF (**a**) and SG (**b**) cell adhesion, as evaluated after 20 min of incubation ((*) for *p* < 0.05 and (**) for *p* < 0.01).

All of the samples allowed cell adhesion. Regarding PF (Figure 4a), notably, on composites, the number of adherent cells was augmented proportionally to the amount of filler. The PCL/ATZ 90/10 supported the lowest number of cellular nuclei compared to PCL/ATZ 80/20 and PCL/ATZ 60/40 (respectively, *p* = 0.03 and *p* = 0.008), which showed increasing numbers of adherent cells. No composite, however, outperformed the PCL in a statistically significant way.

The incorporation of ATZ particles in the polymeric matrix enhanced, instead, the adhesive capabilities of the SG cells (Figure 4b). This result was particularly evident for PCL/ATZ 80/20, in which the highest number of adherent cells was achieved overall in a statistically significant way (*p* < 0.05).

**Figure 5:**
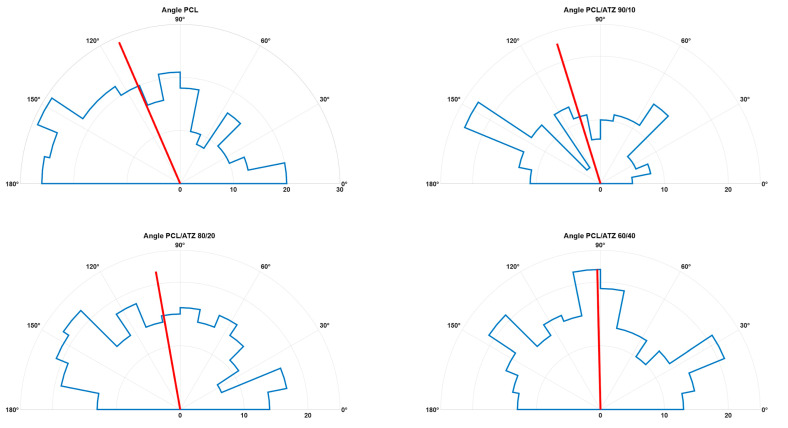
Polar histogram plot of BFE angles measured on each segmented PF cell for all the compounds (in blue are represented the polar bins of the distributions, while in red are highlighted the mean angle values).

The cell spreading analysis using the BFE angle in segmented cells is represented below in a polar histogram plot (Figure 5). The PF spreading, in this case, lost its partial orientation as the ATZ amount was raised inside the PCL matrix. This type of behavior was not confirmed with the SG cells, possibly due to their more rounded shape and their random distributions on surfaces.

**Figure 6:**
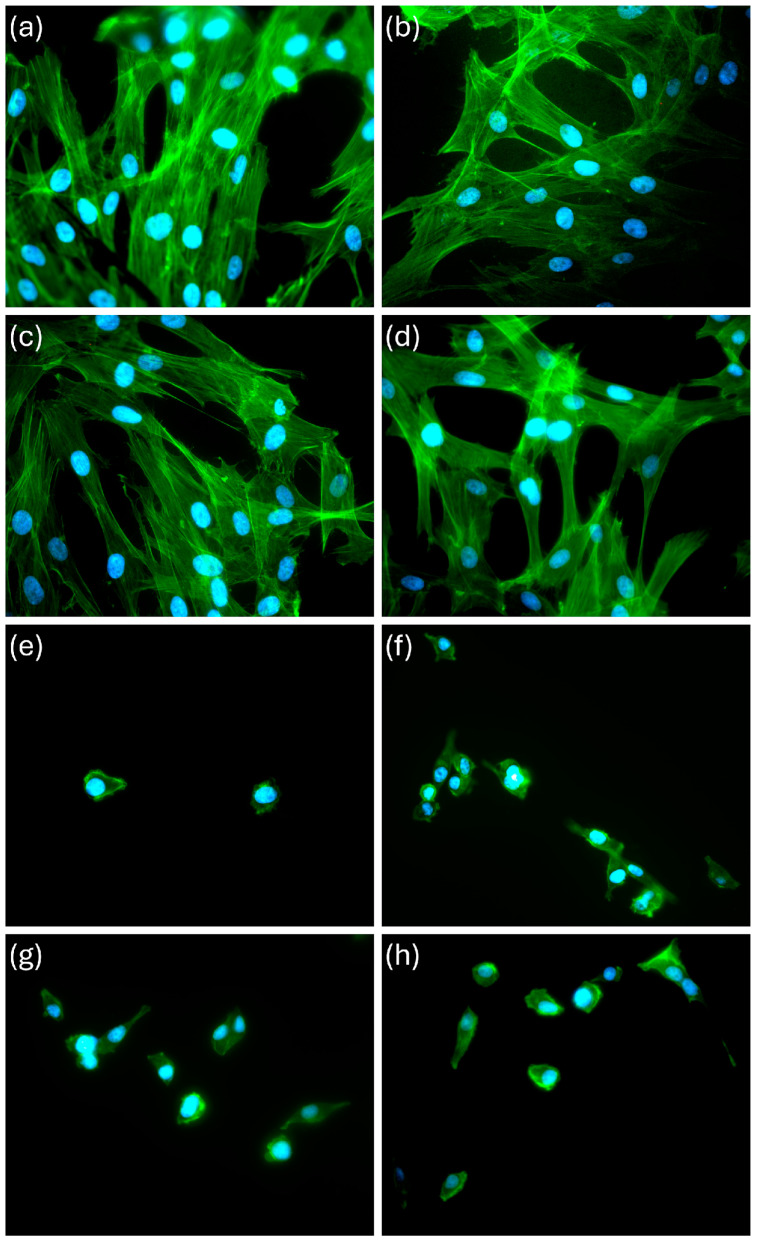
Immunofluorescence microscopy images representing PF cells on (**a**) unmixed PCL, (**b**) PCL/ATZ 90/10, (**c**) PCL/ATZ 80/20, and (**d**) PCL/ATZ 60/40, and SG cells on (**e**) unmixed PCL, (**f**) PCL/ATZ 90/10, (**g**) PCL/ATZ 80/20, and (**h**) PCL/ATZ 60/40, after 24 h of incubation (the nuclei of cells are represented in blue, while the cytoskeletons were stained with green).

Area and perimeter values measured on PF images (Figure 6) were higher for the compounds with ATZ than the unmixed PCL samples. PCL/ATZ 80/20 and PCL/ATZ 60/40 presented the (significant) highest values overall for both the descriptors (*p* < 0.05). Consistent with this, the aspect ratio values were also significantly lower for the pure polymer compared to the others. The complex descriptors of circularity and roundness point out a statistically relevant difference between the PCL/ATZ 90/10 values and the unmixed PCL (*p* = 0.004 and *p* = 0.017). Overall, the total area occupied by the cell accorded with the above-described cell adhesion test (Figure 4a).

PCL/ATZ 80/20 had the (significantly) smallest dimensions of cell spreading for SG in terms of area and perimeter values. The aspect ratio, circularity, and roundness of the cell were, respectively, significantly lower for the first, and higher for the second and third in the PCL samples not filled with ATZ (*p* < 0.05 and *p* < 0.01). The sum of all the area values reported was according to the adhesion test (Figure 4b).

#### 3.5.2. Cell Viability

**Figure 7:**
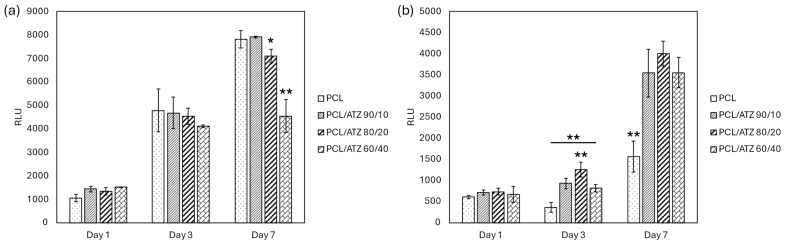
Graph representing PF (**a**) and SG (**b**) cell viability at 1, 3, and 7 days ((*) for *p* < 0.05 and (**) for *p* < 0.01).

At 1 and 3 days, the PF cells grew without significant variations among the different specimens. After 7 days, PF cells proliferated significantly less on PCL/ATZ 60/40 (*p* < 0.01) and PCL/ATZ 80/20 (*p* < 0.05), compared to unmixed PCL and PCL/ATZ 90/10 (Figure 7a).

SG viability showed absolute values lower than those for PF cells. At day 3, SG cells grew significantly better on PCL/ATZ 80/20 compared to the composite materials (*p* < 0.05). At day 7, the growth of SG cells on composite materials was equivalent and significantly higher compared to the unmixed PCL (*p* < 0.01, Figure 7b), suggesting that the filler positively affected the proliferation of SG cells.

## 4. Discussion

The development of an interface material to enhance the mucosal attachment to the implant abutments could limit the probability of peri-implantitis. This work had the purpose of implementing an innovative material, one based on a PCL matrix filled with different amounts of ATZ (10, 20, and 40 wt.%), by using a previously described solvent casting method [25]. After a chemical and physical characterization of the surfaces, these composites were studied as for the early biological response elicited using the two cell types (PF and SG) representative of the mucosal tissue [29,34].

All of the compounds and the unmixed PCL were 3D-printed in regular shapes on the same glass substrate to achieve the smoothest possible surface. This aimed at limiting the variability the additive manufacturing process could have on surface roughness, which is known to play a key role, and may overshadow other features of the bulk material, when characterizing its interface [35,36,37,38]. Nevertheless, SEM micrographs of these surfaces showed the formation of residual stripes of filler that were visible with PCL/ATZ 90/10 and PCL/ATZ 80/20, and oriented parallel to the printing direction. This phenomenon could be due to the swelling ratio of the polymer during the extrusion and cooling process [39,40], which is responsible of changes in volume and viscosity, and gives the filler the tendency to aggregate at the interface between strands. As described by Bellini [36] and Shofner et al. [41,42], the value of the swelling ratio depends on both the material properties and the geometry of the extrusion nozzle. Indeed, inelastic fillers such as ceramics could reduce die swelling, as was also observed here for PCL/ATZ 60/40, with which these stripes were not present, due to the lower ratio of polymer to filler, which allowed a more uniform surface to be obtained.

In this work, the Sz values were augmented proportionally with the composites, consistently with the fact that PCL was expected to be the smoothest surface. Also, PCL/ATZ 60/40 samples showed the highest roughness values (i.e., R_a_, S_a_, R_q_, and S_q_ values). The introduction of ATZ in the polymer matrix affected the S_sk_ of the compounds, ranging from the negative value of PCL/ATZ 90/10 (−1.049) to the positive value of PCL/ATZ 60/40 (1.515), and reaching a value close to 0 for PCL/ATZ 80/20. This means that an asymmetrical distribution of peaks and valleys was present on the two composites with the lowest and the highest amount of ATZ; these had, respectively, a prevalence of valleys and peaks, while a homogeneous distribution thereof characterized PCL/ATZ 80/20. Less predictable outcomes in terms of roughness parameters were achieved for PCL/ATZ 80/20. This compound showed S_a_ values that were half of those from the other samples, and it reached the highest values by far (almost ten times the other specimens) of Kurtosis (S_ku_ = 1400.300 ± 1223.800), which describes very sharp profiles.

According to the wettability assay, all the materials showed a mild hydrophilicity (dH_2_O CA around 67°), with a general preference indicated for lipophilic environments, which is consistent with similar polymeric materials [43]. Furthermore, the filler appeared to amplify this behavior, although a statistically significant difference was achieved only between PCL (CA = 41°) and PCL/ATZ 80/20 (CA = 23°). Overall, this trend was reflected by the moderately higher values of total surface energy (γ) of the PCL (4.15 mN/m) and PCL/ATZ 90/10 (4.14 mN/m), compared to the other two compounds richer in ATZ. These data are in accordance with the high hydrophobicity of ATZ reported by Carvalho et al. [44], as its addition to PCL could enhance the dispersive component of SFE, albeit not significantly. A higher quantity of ATZ also facilitated protein adsorption on the samples’ surfaces, with a three-fold increase found when PCL/ATZ 60/40 (~45 mg/mL) was compared to PCL (~15 mg/mL). Since all the compounds were made of the same PCL matrix, it is reasonable to assume that the amounts of ATZ particles available on the surfaces provided preferrable sites for protein linkage [16], contrary to the findings of Yang et al. [45], in which BSA adsorption on nano-roughened titanium was found to scale linearly with Sq.

Analyzing the early biological response, PF followed the trend of S_sk_ in their adhesion pattern, which is an unprecedented observation, to the authors’ knowledge. On the contrary, neither R_ku_ nor S_ku_ seemed correlated with the PF’s responses to the surfaces tested, which is different from the findings of Frias Martinez et al. [46], in which the samples with higher values of R_ku_ favored the adhesion of human gingival fibroblasts. The importance of Skewness and Kurtosis in orienting cell differentiation was indeed elucidated a decade ago [47] in human mesenchymal cells and osteoblasts. The morphological analysis of cell spreading showed that as the amount of ATZ increased, PF: (a) lost progressively their partial orientation parallel to the printing moving direction, (b) exhibited larger areas, and (c) developed a higher number of filopodia ramifications on the cell borders, compared to PCL. As also confirmed by the values for cell aspect ratio, PCL/ATZ 90/10 and PCL/ATZ 80/20 emerged as the preferable surfaces for PF spreading.

Quite interesting was the correlation between the specific roughness parameters of PCL/ATZ 80/20 and the early cell behavior of SG that adhered the most on this surface in a statistically significant way (Figure 4b). It is conceivable that the topographic pattern of PCL/ATZ 80/20, endowed with low S_a_, high S_ku_, and balanced S_sk_, resulted in the most suitable environment for early adhesion of SG. Unfortunately, the adhesion properties of SG seeded on bio-interfaces, and more generally those of oral keratinocytes, have been poorly studied so far, and the study has been limited to a few materials [48]. Analyzing the morphology of SG, a relevant difference in terms of aspect ratio, circularity, and roundness emerged, owing to their rounded and less spread shape. The surface of unmixed PCL was the case where SG were more rounded in shape. On the contrary, the ATZ particles on the surface appeared to be favorable sites for cell adhesion, although suffering a cell tolerance cap at PCL/ATZ 80/20, since PCL/ATZ 60/40 showed decreasing adhesion-based features. Noteworthily, the favorable effect described on adhesion on PCL/ATZ 80/20 was not obliterated immediately, as this surface still outperformed the other ones, after 3 days, in terms of cell proliferation. At 7 days, instead, all the compounds were remarkably superior to PCL as to cell viability, consistent with Saberian et al. [49]. Unlike SG, PF grew without significant variations among the specimens at 1 and 3 days, but they proliferated significantly less on PCL/ATZ 60/40 (*p* < 0.01) and PCL/ATZ 80/20 (*p* < 0.05), compared to PCL, at day 7. Such a late decrease in the proliferation of PF is unlikely to be due to a lack of biocompatibility, but it is likely ascribable to the presence of differentiative cues. Under this perspective, it is compelling that PCL/ATZ 80/20 could promote the highest number of focal adhesions per cell in PF (Figure S1), resulting the best condition for facilitating early cell adhesion in both the cell models used. Finally, the remarkable divergence concerning the proliferation rates of PF and SG should not be matter of surprise, since cells, depending on their type, may behave quite differently on the same surface [50]. One theoretical explanation of these dissimilar patterns may rely upon the machinery used by PF and SG for interacting with their substrates. While, generally, fibroblasts adhere on a given surface through the formation of classic focal adhesion complexes, epithelial cells lay on and anchor to a basal lamina, requiring specific proteins like laminins, as reported by Riivari et al. [51].

In summary, PCL, when mixed with ATZ, is endowed with certain non-negligible characteristics, such as improved protein adsorption and cell adhesion. Also, the PCL/ATZ compounds could affect cell spreading and the viability of both PF and SG. Specifically, PCL/ATZ 80/20 outperformed all of the other conditions when used as interface for SG and behaved in a manner sufficient to sustain PF viability and adhesion. Hence, such a compound could become a suitable candidate for testing in further studies aiming to functionalize the transmucosal component of dental implants and to improve the mucosal seal, and thus reduce the risk of peri-implantitis.

## Figures and Tables

**Table 1:** Standard parameters of water and di-iodomethane.

Liquid	γ [mN/m]	γ^P^ [mN/m]	γ^D^ [mN/m]
Water	72.8	43.7	29.1
Di-iodomethane	50	2.6	47.4

**Table 2:** Profile roughness R parameters for the different samples measured with the stylus profilometer.

Sample	R_a_ [μm]	R_q_ [μm]	R_sk_	R_ku_	R_z_ [μm]
PCL	0.103 ± 0.019	0.173 ± 0.035	3.273 ± 0.525	40.284 ± 15.079	0.613 ± 0.107
PCL/ATZ 90/10	0.122 ± 0.016	0.234 ± 0.102	−1.597 ± 2.772	85.616 ± 43.422	0.711 ± 0.063
PCL/ATZ 80/20	0.106 ± 0.002	0.143 ± 0.007	−0.644 ± 0.594	10.712 ± 9.004	0.641 ± 0.010
PCL/ATZ 60/40	0.207 ± 0.001	0.306 ± 0.002	−1.003 ± 0.252	11.909 ± 2.799	1.296 ± 0.015

**Table 3:** Areal texture S parameters for the different samples measured with the optical profilometer.

Sample	S_a_ [μm]	S_q_ [μm]	S_sk_	S_ku_	S_z_ [μm]
PCL	0.122 ± 0.021	0.238 ± 0.066	1.825 ± 2.553	27.887 ± 22.966	4.233 ± 2.141
PCL/ATZ 90/10	0.135 ± 0.007	0.227 ± 0.016	−1.049 ± 1.461	91.515 ± 91.120	13.005 ± 6.984
PCL/ATZ 80/20	0.062 ± 0.004	0.102 ± 0.017	−0.123 ± 12.575	1400.300 ± 1223.800	13.861 ± 5.640
PCL/ATZ 60/40	0.155 ± 0.035	0.248 ± 0.057	1.515 ± 5.255	163.070 ± 217.170	14.631 ± 4.138

**Table 4:** Total, polar, and dispersive surface energy of samples, calculated with the OWRK method.

Sample	Surface Energy: Total [mN/m]	Surface Energy: Polar [mN/m]	Surface Energy: Dispersive [mN/m]
PCL	4.15	1.77	2.38
PCL/ATZ 90/10	4.14	1.72	2.43
PCL/ATZ 80/20	4.02	1.48	2.54
PCL/ATZ 60/40	4.09	1.56	2.53

## Data Availability

The original contributions presented in this study are included in the article. Further inquiries can be directed to the corresponding author.

## Supplementary Materials

The following supporting information can be downloaded at: https://www.mdpi.com/article/10.3390/polym16172521/s1, Figure S1. Immunofluorescence microscopy images representing the following: (a) PF cell on unmixed PCL surface; (b) PF cell on PCL/ATZ 80/20 surface (the nuclei of cells are represented in blue, while the cytoskeletons were stained with a green color; red arrows highlight the focal adhesion spots).
