# Supplementary material for: Early Biological Response to Poly(ε-Caprolactone)/Alumina-Toughened Zirconia Composites Obtained by 3D Printing for Peri-Implant Application
Source: Polymers (Basel). 2024 Sep 5;16(17):2521. doi: 10.3390/polym16172521 (PMC11398029; doi:10.3390/polym16172521)
Supplement: Supplementary file 1 [file polymers-16-02521-s001.zip › polymers-3159264-supplementary.pdf]

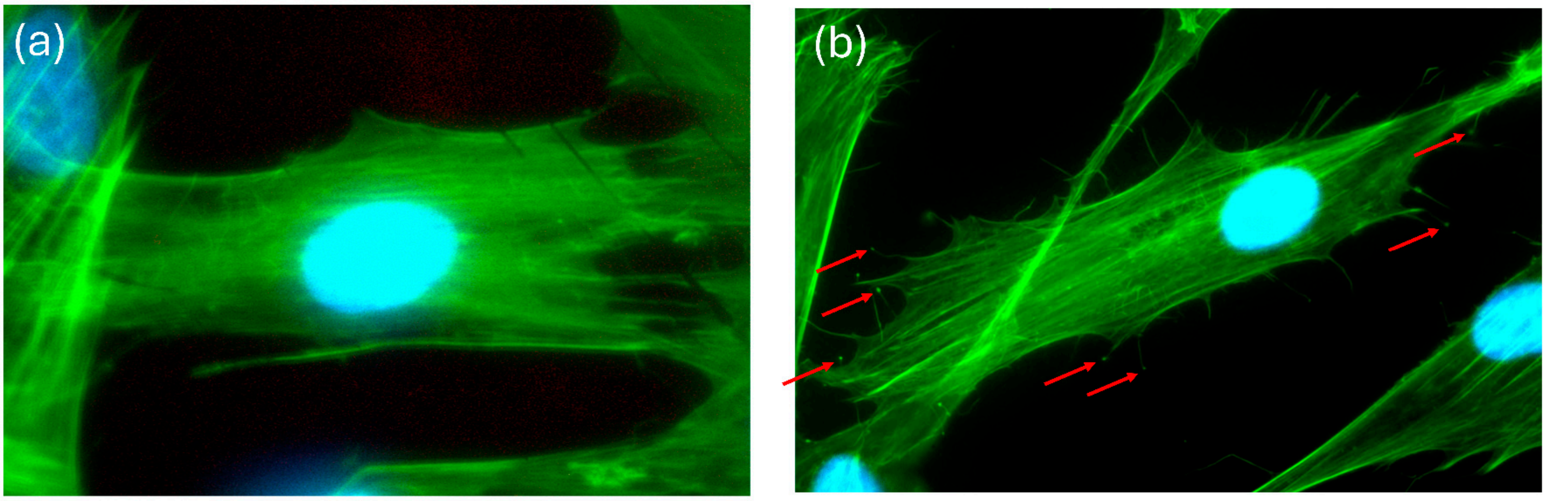

Figure S1. Immunofluorescence microscopy images representing: *a*) PF cell on neat PCL surface. *b*) PF cell on PCL/ATZ 80/20 surface (in blue color are represented the nuclei of cells while the cytoskeleton was stained with green color, red arrows highlighting focal adhesion spots).
